# Increased CSF levels of total Tau in patients with subcortical cerebrovascular pathology and cognitive impairment

**DOI:** 10.1590/1980-57642016dn11-040012

**Published:** 2017

**Authors:** Márcia Radanovic, Florindo Stella, Lis Gomes Silva, Leda L. Talib, Orestes V. Forlenza

**Affiliations:** 1 Laboratório de Neurociencias LIM27, Departamento e Instituto de Psiquiatria, Hospital das Clinicas HCFMUSP, Faculdade de Medicina, Universidade de São Paulo, São Paulo, SP, Brazil.; 2 Biosciences Institute, Universidade Estadual Paulista (UNESP), Campus of Rio Claro, SP, Brazil.

**Keywords:** vascular cognitive impairment, subcortical vascular lesions, cerebrospinal fluid biomarkers, amyloid-β, tau protein, comprometimento cognitivo vascular, lesões vasculares subcorticais, biomarcadores liquóricos, β-amiloide, proteína tau

## Abstract

Cognitive impairment includes mild cognitive decline and dementia, such as Alzheimer's disease (AD) and cerebrovascular-related pathologies.

**Objective:**

To investigate the profile of AD-related CSF biomarkers in a sample of cognitively impaired and unimpaired older adults with concomitant subcortical cerebrovascular burden.

**Methods:**

Seventy-eight older adults attending an outpatient psychogeriatric clinic were enrolled. Diagnoses were based on clinical, neuropsychological, laboratory, and neuroimaging data. Participants were classified into: cognitively normal (controls, n = 30), mild cognitive impairment (MCI, n = 34), and dementia (AD, n = 14). All subjects were submitted to CSF analyses for determination of amyloid-beta (Aβ_1-42_), total tau (t-tau), phosphorylated tau (p-tau) and Aβ_1-42_/p-tau ratio according to the Luminex method. MRI was performed in all individuals, and was scored independently by two experts according to Fazekas scale. Statistical analyses were conducted with the aid of general linear model procedures, and the Chi-squared test.

**Results:**

T-tau levels were significantly associated with subcortical lesion pattern when Fazekas was considered as a group factor. CSF biomarkers were not associated with MCI, AD, or controls when considered separately. There was a tendency for reduction in CSF Aβ_1-42_ together with increasing Fazekas scores, but without statistical significance. Comparisons of Aβ_1-42_ and t-tau with each clinical group or with each neuroimaging pattern did not reach statistical differences. Likewise, Fazekas scores had no impact on CAMCOG scores.

**Conclusion:**

We found a significant association between t-tau levels and subcortical lesions when all Fazekas classifications were considered as a single group; comparisons of Fazekas subgroups and CSF biomarkers did not reach significance.

## RESUMO.

O comprometimento cognitivo inclui alterações leves da cognição e demência, como doença de Alzheimer (DA) e patologias vasculares associadas.

**Objetivo:**

Investigar o perfil de biomarcadores da DA no líquor e doença cerebrovascular concomitante em idosos com e sem alterações cognitivas.

**Métodos:**

Foram incluídos 78 sujeitos de um ambulatório de psicogeriatria. Efetuaram-se os diagnósticos com base em dados clínicos, neuropsicológicos, laboratoriais e neuroimagem. Os participantes foram classificados em: cognitivamente normais (controles, n = 30), comprometimento cognitivo leve (CCL, n = 34) e demência (DA, n = 14). Todos foram submetidos ao exame liquórico para determinação de β-amiloide (Aβ_1-42_), tau total (t-tau), tau fosforilada (p-tau) e razão Aβ_1-42_/p-tau, segundo o método de Luminex. RM foi efetuada em todos os indivíduos. Dois especialistas independentes avaliaram as imagens segundo a escala de Fazekas. As análises estatísticas basearam-se em modelo linear geral e teste qui-quadrado.

**Resultados:**

T-tau foi significantemente associada ao padrão de lesão subcortical quando o grau de Fazekas foi considerado como fator grupal. Não houve associação entre biomarcadores e diagnóstico clínico de CCL, DA e grupo controle, considerados individualmente. Observou-se uma tendência de redução de Aβ_1-42_ concomitante com elevação dos escores de Fazekas, sem correlação significante. Comparações entre Aβ_1-42_ e tau e diagnóstico clínico ou neuroimagem não foram significantes. Os resultados de Fazekas não influenciaram os escores do CAMCOG.

**Conclusão:**

Como principal resultado, observou-se associação significante entre os níveis de t-tau e lesões subcorticais quando as classificações de Fazekas foram incluídas em um único grupo. As comparações dos subgrupos de Fazekas e biomarcadores liquóricos não foram significantes.

## INTRODUCTION

Mild cognitive impairment (MCI) is a clinically and pathologically heterogeneous entity with distinct characteristics related to particular cognitive changes, velocity and magnitude of clinical deterioration, time period of stability, or time to conversion to a specific dementia, or recovery of normal cognition. Vascular cognitive impairment is a broad-spectrum concept that comprises distinct levels of clinical changes from mild manifestations of cognitive decline to established dementia; it also encompasses mixed entities such as vascular Alzheimer's disease (AD)-related pathologies.^1^

Rates per year of conversion from MCI to Alzheimer's disease (AD) vary widely. A Brazilian investigation identified a 6% annual conversion,^2^ differing from rates found in the original Petersen's study (10 to 15%),^3^ and from those described by other groups.^4^^,^ 5 Furthermore, individuals with MCI who control cerebrovascular risk factors through a healthy life-style, cognitive resilience and appropriate mental health care, tend to remain cognitively stable, or attain reversion to normal cognition.^6^^,^ 7

Overall prevalence of cerebrovascular lesions tends to be higher with aging. Thus, individuals aged 60-69 years had a frequency of 17.8% of these cerebrovascular events, whereas the proportion reached 38.3% in individuals over 80 years; furthermore, strictly lobar microbleeds were significantly more frequent among APOE e4 carriers than noncarriers.^8^ In addition to underlying neurodegenerative mechanisms, cerebrovascular disease has been considered an important etiological constituent, associated with a more aggressive clinical course of MCI.^9^^,^ 10 Microbleeds can reflect damaged microvasculature, leading to decrease in blood flow and subsequent ischemia with probable tau pathology and neuronal degeneration.^11^ Ischemic lesions are detectable as subcortical hyperintensities using fluid-attenuated inversion recovery (FLAIR) of the magnetic resonance (MRI) technique. Subcortical hyperintensities are very common in vascular dementia and aging, but also in AD, and have been related to impaired cognition, particularly executive functions.^12^^,^ 13

Regarding the cerebral distribution of cerebrovascular events, a study supports the hypothesis that strictly lobar microbleeds are associated with amyloid angiopathy,^8^^,^ 12 while these vascular events located in deep gray matter or in infratentorial structures emerge from hypertension, diabetes, hypercholesterolemia, and may lead to atherosclerotic microangiopathy.^8^^,^ 11

A comprehensive study from the Alzheimer's Disease Neuroimaging Initiative (ADNI) investigated the occurrence of cerebrovascular lesions in patients with MCI, AD, and in cognitively preserved individuals.^11^ Neither diagnostic group was statistically different concerning these vascular occurrences. The authors identified that patients having at least three microbleeds in brain lobar tissue had lower levels of CSF Aβ_1-42_ according to statistical regression model analyses.^11^ These findings may reflect a cerebral amyloidosis dose-response association. On the other hand, the investigation failed to find similar statistical significance in patients presenting cerebrovascular lesions in deep gray matter or in infratentorial structures. Another interesting result of the study was the association between cerebrovascular events and phosphorylated tau (p-tau). Using logistic regression, the authors detected that patients having at least one lobar vascular lesion had more than double the odds of presenting abnormal levels of CSF p-tau. However, unlike amyloid-β, the authors did not observe a dose-response association between CSF p-tau levels and lobar vascular lesions. Moreover, gray or infratentorial vascular lesions were not statistically correlated with CSF p-tau levels.^11^

A study investigating the risk of progression from MCI to AD used a methodological strategy combining regional cerebral blood flow with cerebrospinal fluid biomarkers.^14^ Patients were followed at least until they converted to dementia or until they remained cognitively stable for more than 4 years, during a period of 5.2 years on average. MCI patients with both decreased parietal blood flow and pathological levels of cerebrospinal tau and CSF Aβ_1-42_ were at very high risk of subsequent progression to AD, as well as a further more accelerated neurodegenerative process.^14^

Determining a biomarker profile able to discriminate those individuals at risk of progression from MCI to AD is crucial for improving accuracy of diagnostic strategies.^10^ In this scenario, patients with MCI and simultaneous cerebrovascular disease are at increased risk of developing AD.^4^

The main purpose of the present study was to compare CSF Aβ_1-42_ levels and t-tau and p-tau concentrations with the degree of cerebrovascular lesions according to the Fazekas scale, in patients with MCI and AD, and in cognitively preserved subjects.

## METHODS

Seventy-eight elderly attending an outpatient psychogeriatric clinic of a university hospital were enrolled in this study. All subjects were assessed for cognitive status by the Brazilian version of the Cambridge Cognition Examination (CAMCOG), a subscale from the Cambridge Examination for Mental Disorders of the Elderly (CAMDEX) interview^15^^,^ 16 and full neuropsychological examination including the Rivermead Behavioural Memory Test,^17^^,^ 18 the Fuld Object-Memory Evaluation,^19^^,^ 20 the Trail Making Test A and B,^21^^,^ 22 the Short Cognitive Test,^23^^,^ 24 and the Wechsler Adult Intelligence Scale-Revised (WAIS-R) Vocabulary and Block Design subtests.^25^^,^ 26 These tests were applied for cognitive assessment and diagnosis as a gold-standard procedure. The CAMCOG was considered in this study as a reference for the cognitive status of each subgroup, as its psychometric properties meet the requirements for such discrimination.^2^ The 21-item Hamilton Depression Scale (HAM-D)^27^^,^ 28 was administered to rule out depressive comorbidity with euthymia defined as a score ≤ 7. Functional status was assessed using the Informant Questionnaire on Cognitive Decline in the Elderly (IQCODE),^29^^,^ 30 with a cut-off score of 3.41 being used to discriminate dementia from MCI and normal function. Laboratory examinations included complete blood count and chemistry serum levels, syphilis test, thyroid function, folic acid and vitamin B12, and blood lipid profile. A multidisciplinary team established all diagnoses based on clinical, neuropsychological, laboratory and neuroimaging data. Subjects were grouped into controls (30 subjects), MCI (according to Petersen's criteria – 34 subjects),^31^ and AD (according to the NIA-AA criteria – 14 subjects).^32^ All subjects were submitted to lumbar puncture and had their cerebrospinal fluid levels of Aβ_1-42_ peptide, total tau (t-tau), phosphorylated tau (p-tau), and Aβ_1-42_/p-tau ratio determined using the INNo-Bia AlzBio3 assay (Innogenetics, Ghent, Belgium), a multiplex microsphere-based LuminexxMAP platform that allows simultaneous analysis of these biomarkers. MRI brain scans were performed in all participants and were scored by two neurologists according to the Fazekas scale^33^ based on axial fluid-attenuated inversion recovery (FLAIR) acquisitions. CSF biomarker levels and Fazekas classification were not taken into account for subjects' diagnoses.

Statistical analysis. Intergroup comparisons of age, education, CAMCOG scores, and CSF biomarkers levels were analyzed using a series of one-way analyses of variance (ANOVAs) followed by the Scheffé post-hoc procedure to test for difference between means. The Chi-squared test was used to compare intergroup distribution for sex and Fazekas classification. General linear model (GLM)-based analyses were used to verify the influence of diagnostic status, biomarker levels, Fazekas classification, and age on CAMCOG scores.

## RESULTS

Demographic and clinical data for the sample are displayed in Table 1.

**Table 1:** Demographic and cognitive data by diagnostic subgroup.

Demographic and cognitive assessment		Controls	MCI	AD	p-value
N (%)		30 (38.5)	34 (43.6)	14 (17.9)	
Age		70.5 (4.7)	71.8 (4.8)	76.0 (7.4)	0.070
Education		12.5 (6.0)	10.6 (5.5)	8.0 (4.5)	0.048 Controls ≠ AD
Sex*	M	8	9	5	0.819
	F	22	23	9	
CAMCOG		90.2 (13.0)	86.9 (12.0)	74.2 (14.1)	0.001 AD ≠ controls & MCI

One-way ANOVA with Scheffé post-test;

* * Chi-squared test. AD: Alzheimer's disease; MCI: mild cognitive impairment; M: male; F: female; CAMCOG: the Cambridge Cognition Examination.

The distribution of biological data such as CSF biomarkers and deep white matter (DWM) hyperintensities, as measured by Fazekas scale, among diagnostic groups is given in Table 2.

**Table 2:** Biomarkers data by diagnostic subgroup.

CSF biomarkers and Fazekas	Controls	MCI	AD	p-value
Aβ_1-42_ pg/mL	426.2 (165.2)	423.2 (145.7)	374.9 (123.6)	0.535
P-tau pg/mL	46.8 (26.5)	50.1 (32.4)	48.3 (20.3)	0.896
T-tau pg/mL	85.6 (50.3)	107.1 (78.9)	127.2 (79.3)	0.179
Aβ_1-42_/p-tau	11.2 (6.3)	11.7 (7.9)	9.0 (5.8)	0.462
**Fazekas (N)**	** **	** **	** **	**Frequency (%)**
0	1	5	0	6 (7.7)
1	17	20	7	44 (56.4)
2	9	8	4	21 (26.9)
3	3	1	3	7 (9.0)

One-way ANOVA with Scheffé post-test;

* Chi-squared test. AD: Alzheimer's disease; MCI: mild cognitive impairment; CSF: cerebrospinal fluid; Aβ: amyloid-β; P-tau: phosphorylated tau; T-tau: total tau.

ANCOVA and multiple regression procedures showed that “diagnosis” (F = 4.471, p = 0.015) and “tau levels” (F = 5.791, p = 0.019) had an influence on CAMCOG scores. Fazekas grade did not impact CAMCOG scores in any diagnostic group (F = 0.403, p = 0.752 for controls; F = 0.141, p = 0.935 for MCI; F = 3.984, p = 0.053 for AD). Age and CSF levels of Aβ_1-42_, p-tau or Aβ_1-42_/p-tau levels also had no association with CAMCOG scores (all p > 0.05). An ANOVA procedure using CSF biomarker levels as the dependent variable and Fazekas grade as the group factor showed that Tau-levels were associated with degree of DWM (Table 3).

**Table 3:** CSF biomarker levels according to Fazekas grade.

Variable	Fazekas = 0 (6)	Fazekas = 1 (44)	Fazekas = 2 (21)	Fazekas = 3 (7)	p-value
Aβ_1-42_ pg/mL	444.4 (130.7)	428.9 (157.1)	395.9 (141.6)	367.5 (149.3)	0.653
P-tau pg/mL	41.6 (18.6)	49.5 (28.5)	42.5 (19.6)	66.3 (46.2)	0.246
T-tau pg/mL	82.8 (30.8)	105.3 (75.9)	80.0 (30.9)	165.2 (102.5	**0.038** 3 ≠ 1 & 2 (p<0.05)
Aβ_1-42_/p-tau	13.0 (8.1)	11.0 (6.4)	11.5 (7.9)	7.6 (6.5)	0.515

CSF: cerebrospinal fluid; Aβ: amyloid-β; P-tau: phosphorylated tau; T-tau: total tau.

AD patients were less educated than both controls and MCI patients and performed worse on the CAMCOG, as expected. Although not reaching statistical significance, there was a trend towards AD patients having lower CSF levels of Aβ_1-42_ and higher levels of t-tau, along with lower Aβ_1-42_/p-tau ratios.

The distribution of deep white matter (DWM) hyperintensities, as measured by the Fazekas scale, among diagnostic groups is displayed in Table 3. Most subjects (56.4%) were classified as Fazekas 1 (punctate foci) regardless of diagnosis, but there was no relationship between diagnosis and Fazekas classification (Chi-square = 8.229, p = 0.221).

## DISCUSSION

In the present study we addressed the profile of AD-related CSF biomarkers in the presence of concomitant subcortical vascular abnormalities in a cross-section of older adults with varying degrees of cognitive impairment recruited from a specialized memory clinic. Regarding tau levels, our data suggest an association between subcortical lesions and tau levels. Increased total tau levels were significantly associated with the degree of DWM changes when Fazekas grade was considered as a group factor, i.e., when all individuals were aggregated into a single group, independently of subcortical lesion pattern. We also detected a progressive reduction in levels of Aβ_1-42_ as the degree of DWM increased, i.e., from Fazekas = 0 (444.4 pg/mL) to Fazekas = 3 (367.5 pg/mL). However, differences between biomarker groups and Fazekas scores were not statistically significant. As expected, our study showed a trend towards AD having lower CSF concentrations of Aβ_1-42_ and higher levels of t-tau, together with lower Aβ_1-42_/p-tau ratio. Regarding MRI brain scans, most subjects were classified as Fazekas 1. Once more, we found no statistically significant differences between levels of tau or Aβ_1-42_ and isolated neuroimaging group of Fazekas, or between biomarkers and individual clinical diagnosis. In addition, Fazekas grade did not exert an impact on CAMCOG scores in any diagnostic group.

In contrast with our data, some cross-sectional studies have demonstrated an increased deterioration of global cognition or executive dysfunctions in patients with cerebrovascular disease,^12^ as well as in patients with lesions located strictly in lobar brain regions.^34^ Even community-dwelling older adults showed subnormal cognitive performance associated with cerebrovascular disease.^35^

Neuropsychological assessments in patients with AD sharing cerebrovascular events tend to show worse cognitive impairment than evaluations among those with “pure” AD.^11^^,^ 36 Thus, patients with multiple microbleeds having lower CSF amyloid-β and higher t-tau and p-tau exhibited worse performance on cognitive assessments for global functions (Mini-Mental State Examination), object naming (Visual Association Test), language (Verbal Fluency Test), and digit span (forward and backward) than those without microbleeds.^37^

An intriguing issue is the impact of cerebrovascular disease on cognitive performance over time in patients with CSF abnormalities. The above-mentioned study^11^ used a linear mixed-effects model to analyze the association between vascular events and cognition, adjusting for some covariates including CSF Aβ_1-42_ levels and diagnostic group. Patients having at least one lobar vascular event had a 1.4 point decline per year on the Alzheimer's Disease Assessment Scale – cognitive subscale (ADAS-Cog) when compared with those free from lobar vascular lesions, who showed a 0.8 point decline during the same period. In addition, cognitive decline reached 2.3 points per year on the scale among patients who had more than three lobar vascular lesions. Cognitive impairment persisted even after adjusting for CSF Aβ_1-42_ levels. The authors suggested that these cerebrovascular events *per se* might reflect underlying amyloid angiopathy and contribute to cognitive deterioration.

Furthermore, longitudinal investigations involving individuals with cerebrovascular disease found worse cognitive global deterioration,^38^ a progressive decline in memory impairment,^39^ an increased rate of conversion from MCI to AD,^40^ as well as an elevated risk of developing incident dementia.^41^

Acute stroke also seems to alter levels of CSF Aβ_1-42_ and tau. An analysis of these biomarkers found increased levels of CSF tau among patients suffering from acute ischemic events detected by combined clinical and MRI assessments.^9^ CSF p-tau levels were similar between individuals with MCI and those with stroke, suggesting some neurodegeneration within both groups, although this biomarker was more altered in AD. Furthermore, CSF Aβ_1-42_ levels were low among stroke patients, as extensively described in AD.^9^ These findings suggest that changes in the CSF biomarker pattern can be caused by ischemic events themselves, independently of the neurodegenerative process alone.

Regarding molecular neuroimaging and brain perfusion imaging, Bangen et al.^42^ found that older adults with positive Aβ deposition, as measured by retention of florbetapir in cortical regions, had higher cerebral blood flow. Increased cerebral perfusion in positive Aβ deposition involved cortical regions mainly affected in AD, such as the hippocampus, posterior cingulate, and precuneus. Cognitively, these patients had poorer verbal memory. This hyperperfusion has been interpreted as indicating cellular and vascular compensatory activity in response to pathologic damage related to early stages of AD, notably in APOE e4 carriers,^43^^-^ 45 and reflects a biological strategy to counteract memory challenges.^44^ Conversely, among negative Aβ load patients there was no significant association between resting cerebral blood flow and global cognition, notwithstanding a trend toward higher perfusion in the posterior cingulate and better performance on verbal memory tasks.^42^ Interestingly, hyperperfusion in early MCI tends toward hypoperfusion later in MCI, and exacerbates when approaching clinical diagnosis of dementia.^42^

A complex interaction of multiple mechanisms underlying the neurodegenerative process in AD has been recognized, even at early stages of the disease, and includes cerebrovascular events, molecular changes, and microstructural disorder; this phenomenon predicts brain atrophy and has an unfavourable impact on cognition and behaviour.^12^^,^ 13 ^,^ 17 Therefore, damaged microvasculature may lead to reduced blood flow into brain regions, subsequently causing ischemia, tau pathology, and neuronal degeneration.^11^ Disruption of cerebral blood vessels and disorder of the hemodynamic system induce a higher risk of developing AD in patients with MCI who present pathological levels of cerebrospinal fluid biomarkers.^14^ Decreased cerebral blood flow can elicit synaptic loss and neuronal death in cortical structures.^46^^,^ 47 Likewise, a post-mortem investigation demonstrated that p-tau labelling was more intense around arteries and arteriole vessels with Aβ deposition than around non-Aβ-laden vessels.^48^ Nevertheless, it is assumed that tau-related AD pathology and vascular periventricular lesions constitute two distinct interactive etiologies of MCI that aggravate neuronal vulnerability.

Although subcortical hyperintensities have been associated with more pronounced pathological CSF biomarkers, cognitive decline could not be attributed to cerebrovascular disease *per se*.^37^ Level of education, longer disease duration, and more severe cerebral atrophy may contribute to the clinical course, although each isolated variable can only partially explain the cognitive worsening. Moreover, the reciprocal interference of cerebrovascular disease and AD downstream evolution warrants a more in-depth approach that highlights the magnitude of each biological mechanism and its impact on cognition.

In conclusion, in our study tau levels were associated with degree of DWM when all individuals were aggregated into a single group, independently of clinical diagnosis. There was a progressive reduction in levels of Aβ_1-42_ as degree of DWM increased, although without statistical significance. Again, there were no differences between levels of tau or Aβ and isolated neuroimaging group of Fazekas, or between biomarkers and individual clinical diagnosis. The small number of subjects, especially in the AD group, at least in part, could explain the absence of statistical differences between diagnosis groups regarding Fazekas scores and biomarker concentrations. Despite the limitation concerning the small sample, the fact that all participants, including the control group, underwent MRI and especially CSF exams, represents a strength of this study. Future studies should explore the current methodological strategy in a large sample, where this may provide more consistent data.
